# BMP and TGFβ use and release in bone regeneration

**DOI:** 10.3906/sag-2003-127

**Published:** 2020-11-03

**Authors:** Zeynep BAL, Junichi KUSHIOKA, Joe KODAMA, Takashi KAITO, Hideki YOSHIKAWA, Petek KORKUSUZ, Feza KORKUSUZ

**Affiliations:** 1 Department of Orthopaedic Surgery, Graduate School of Medicine, Osaka University, Osaka Japan; 2 Department of Histology and Embryology, Medical Faculty, Hacettepe University, Ankara Turkey; 3 Department of Sports Medicine, Medical Faculty, Hacettepe University, Ankara Turkey

**Keywords:** BMP, TGFβ, bone regeneration, release, signaling, cytokine

## Abstract

A fracture that does not unite in nine months is defined as nonunion. Nonunion is common in fragmented fractures and large bone defects where vascularization is impaired. The distal third of the tibia, the scaphoid bone or the talus fractures are furthermore prone to nonunion. Open fractures and spinal fusion cases also need special monitoring for healing. Bone tissue regeneration can be attained by autografts, allografts, xenografts and synthetic materials, however their limited availability and the increased surgical time as well as the donor site morbidity of autograft use, and lower probability of success, increased costs and disease transmission and immunological reaction probability of allografts oblige us to find better solutions and new grafts to overcome the cons. A proper biomaterial for regeneration should be osteoinductive, osteoconductive, biocompatible and mechanically suitable. Cytokine therapy, where growth factors are introduced either exogenously or triggered endogenously, is one of the commonly used method in bone tissue engineering. Transforming growth factor β (TGFβ) superfamily, which can be divided structurally into two groups as bone morphogenetic proteins (BMPs), growth differentiation factors (GDFs) and TGFβ, activin, Nodal branch, Mullerian hormone, are known to be produced by osteoblasts and other bone cells and present already in bone matrix abundantly, to take roles in bone homeostasis. BMP family, as the biggest subfamily of TGFβ superfamily, is also reported to be the most effective growth factors in bone and development, which makes them one of the most popular cytokines used in bone regeneration. Complications depending on the excess use of growth factors, and pleiotropic functions of BMPs are however the main reasons of why they should be approached with care. In this review, the Smad dependent signaling pathways of TGFβ and BMP families and their relations and the applications in preclinical and clinical studies will be briefly summarized.

## 1. Introduction

Trauma, tumor resection, degeneration, deformation may cause bone defects in which the healing process is controlled by cytokines as bone morphogenetic proteins (BMP) or transforming growth factor beta (TGFβ), are a great challenge for regeneration studies [1,2,3,4]. Bone loss is frequently difficult to reconstruct due to limitations in autografts, allografts, natural or synthetic composites [1]. In 2015 Sheikh et al. [5] mentioned that about 5% to 10% of all the procedures related with impaired healing are due to physiological stress and they cause morbidity in patients while a significant economic loss is recorded. Every year at least 6.3 million people are reported to suffer from bone fractures in the US as reported by the AAOS and 25% of these cases require bone grafting [5]. Autografts, standard in treatment of bone defects, are the most commonly used grafts because of their osteoinductivity, osteoconductivity and being nonimmunogenic; however, they have disadvantages as increased surgical time and donor site morbidity [1,2,4,6,7]. On the other hand, allografts are also osteoconductive and slightly osteoinductive and do not have complications as donor site morbidity but have the probability of disease transmission or rejection by the immune system and their bone repair capability is half of the autografts, with higher price [1,4,8,9]. Together with these disadvantages, the collected tissue for regeneration may not always be sufficient enough to fill the defect [10,11]. In order to overcome these constrictions, synthetic materials and composites became a popular research area in tissue engineering field. 

An optimal graft should be osteoinductive, osteoconductive, nonimmunogenic, biocompatible, mechanically compatible and depending on the application area and purpose, it may be biodegradable or nonbiodegradable [11,12]. The new generation of smart materials are aimed to achieve bone regeneration with the lowest possible use of cytokines in order to decrease the side effects of these growth factors [13]. Success of the implant also depends on the induction of migration capability of cells to implant area and in the presence of BMP, BMSCs are reported to migrate to the regeneration area [14]. In the process of regeneration, cell migration to damaged areas, angiogenesis, fibrosis, chemokine secretions and differentiation of stem cells are especially important [15]. Thus, osteogenic growth factors or cytokines are generally used in bone tissue engineering due to their osteoinductive abilities because they are able to attract the progenitor cells to the defect area and promote the cell proliferation, migration and endogenous repair mechanisms [1,10]. Their effects on the mechanism of bone regeneration via migration, proliferation, differentiation and reconstruction of the extracellular matrix are in time dependent manner [1,4]. 

Throughout these cytokines, bone morphogenetic proteins (BMPs), as being the biggest subfamily of TGFβ superfamily, are the most commonly used growth factors because they are already present in bone tissue and reported to be necessary for fetal tissue development and fracture repair [1,2,4,6,10,16]. TGFβ, itself, is also known as an important growth factor in regulation of osteoblast and osteoclast activities which is an important process for bone homeostasis and remodeling [17,18]. 

The use of BMP-2 in spine for anterior lumbar spinal fusion has approved by FDA (US Food and Drug Administration), yet the excess amount of BMP, leakage or uncontrolled burst release can cause inflammation, edema, nerve root compression and seroma formation, radiculitis, ectopic bone formation, immune response, osteolysis, cervical and soft tissue edema, osteoclast mediated bone resorption, wound complications, urogenital disorders, inappropriate adipogenesis and heterotrophic bone formation [1,2,11,19,20]. Although BMPs are known as key factors in the commitment of stem cells to osteoprogenitor lineage, especially in the spinal applications, the excess amount of BMP is reported to cause cancer [11]. Effects of BMPs on growing tissues are also unclear, so while using BMPs during childhood, adolescence and pregnancy extreme care should be taken and its use in patients with tumor and active infections or pregnancy is reported to be contraindicated [1,21]. 

## 2. TGFβ superfamily

**Figure 1:**
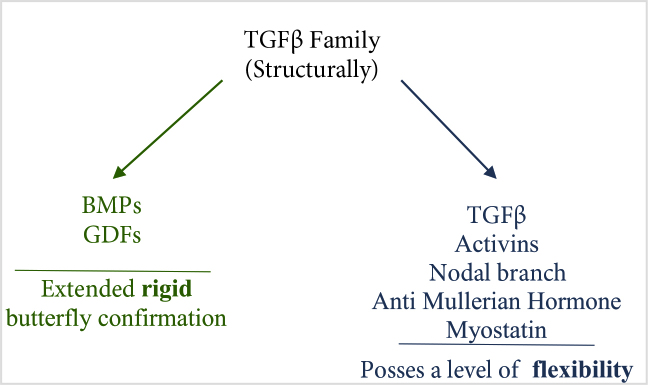
TGFβ superfamily members.

IGFβ, TGFβ and BMP families are subfamilies of TGFβ superfamily. TGFβ superfamily members are known to be produced by osteoblasts (OB) and other bone cells and they are found in bone matrix abundantly [22–24]. They are also known to stimulate osteoblast proliferation and differentiation in vitro, bone formation when externally administrated in vivo, and cell migration-survival-differentiation, osteoprogenitor proliferation, early differentiation, osteoblastic lineage commitment through noncanonical MAPK and canonical Smad pathways [18, 23–27]. TGFβ family structurally can be divided into two as, BMPs together with GDFs which have a rigid butterfly confirmation and activins, Nodal branch, anti-Mullerian hormone, Myostatin with TGFβ which possess a level of flexibility [22, 28, 29] (Figure 1). 

**Figure 2:**
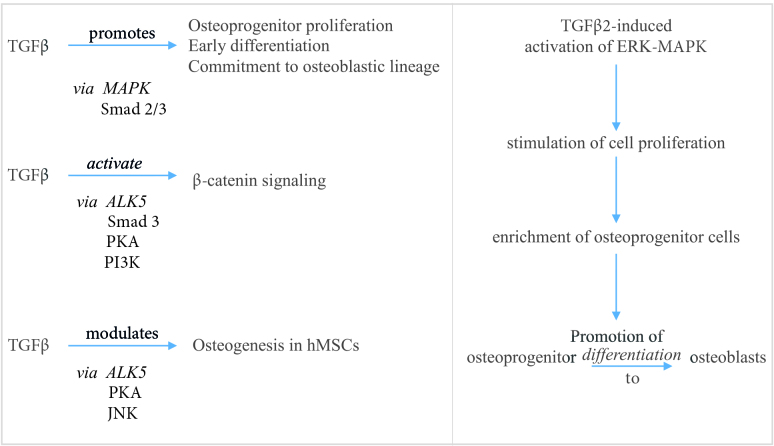
Mechanisms of TGFβ on bone.

In cell signaling, TGFβ signals are conveyed through 2 types of serine-threonine kinase transmembrane receptors, as type II and type I Receptors, to Smad molecules, which are intracellular mediators of this signaling pathway or in other words they are mostly “one-way buses” of the TGFβ signaling pathway that carry signals between nucleus and cytoplasm and this signaling system is highly conserved through evolution [22, 25–33]. The signaling system that uses these one-way Smad buses are known as Smad dependent or canonical TGFβ signaling system [26,27,29]. The other pathway is known as non-Smad pathway which includes p38 - MAPK, Rho - like GTPase signaling and PI3K/AKT pathways [27,28]. As an example of how noncanonical TGFβ pathways works; TGFβ can promote osteoprogenitor proliferation and osteoblastic lineage commitment via MAPK-Smad2/3, also TGFβ2 can induce the activation of ERK-MAPK and can promote osteoblastic differentiation via stimulating cell proliferation; or via MAPK pathways BMP induced OB differentiation can be induced [26,27,33] (Figure 2). 

**Figure 3:**
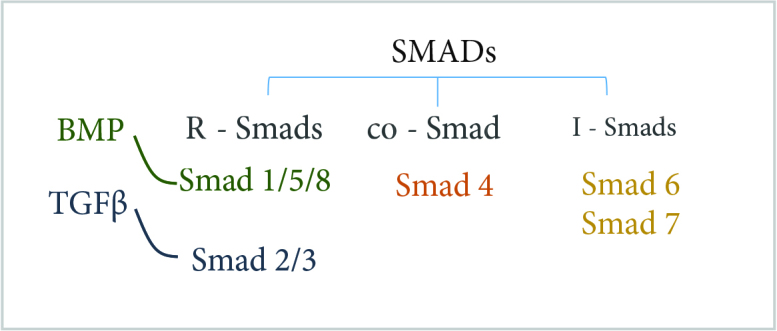
Smad types.

**Figure 4:**
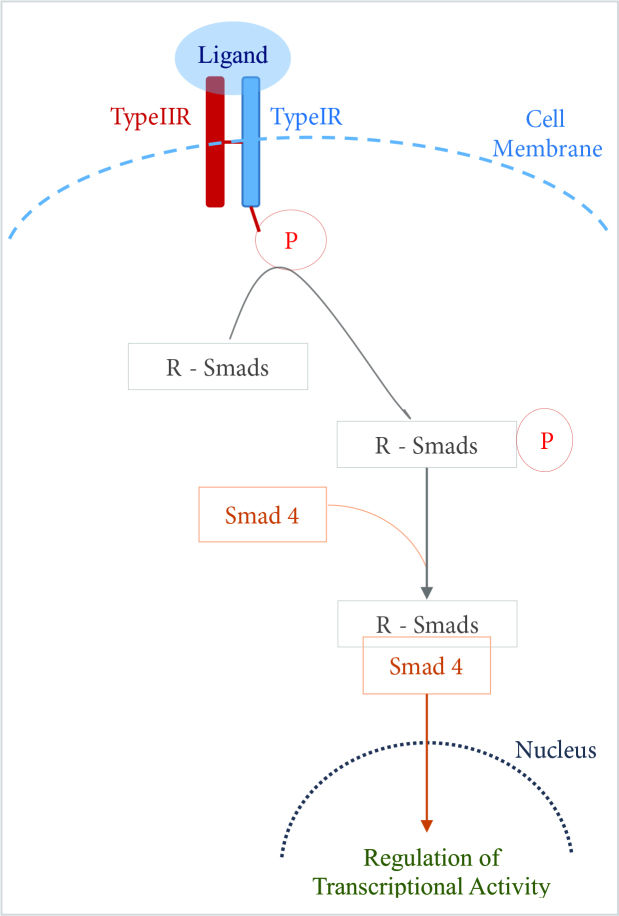
Basic mechanism of signal transduction via Smad dependent pathway in TGFβ family signaling.

In the canonical TGFβ pathway, the Smad molecules are the main intracellular mediators. In mammals, 8 Smad molecules from Smad1 to Smad8 were defined. They are categorized as (receptor regulated-Smads, the downstream key molecules for receptors to transduce signals) R-Smads, (common mediator-Smad) Co-Smad and (inhibitory-Smads) I-Smads [26, 28, 31, 33, 34– 39] (Figure 3). Smad 1, Smad 2, Smad 3, Smad 5, Smad 8 are known as R-Smads, Smad 4 is known as Co-Smad, and Smad 6, Smad 7 are known as I-Smads [28, 29, 32–34]. Briefly, transphosphorylation of type I-R (type I Receptor) by constitutively active type II-R (type II Receptor) kinase happens via ligand (BMPs or TGFβs) binding. The activation of type I serine/theronine kinases (type I-R) initiates the post receptor signaling cascade via phosphorylation of R-Smads. Phosphorylated R-Smads forms a complex with Co-Smad and this complex formation translocates R-Smad/Co-Smad complex to the nucleus, where they can either integrate with DNA binding proteins or directly regulate transcriptional activity by binding to regulatory elements of target genes either as monomers or in association with Co-Smad [22, 23, 28, 30, 33–35] (Figure 4). 

Rahman et al. [28] mentioned that in nucleus Smads are also able to participate in histone modifications and/or chromatin remodeling. I-Smads on the other hand, negatively regulates this signaling process by preventing the complex formation between R-Smads and Co-Smad via blocking the phosphorylation of R-Smads [32] or as for Smad 6, competing with R-Smads for Smad4 (Co-Smad) binding and forming a nonfunctional complex with Smad4 [26]. Smurfs, as Smurf1 and Smurf2, are also reported to suppress TGFβ or BMP signaling via degradation of Smads and receptors for TGFβ and BMP [26]. 

Although TGFβ contributes to bone regeneration also via potentiating the osteoinductive activities of BMP, osteoinductive activity of TGFβ is reported to be much lower than BMPs [24]. Thus in bone regeneration studies TGFβ family and especially BMP family are one of the most commonly used growth factors. For this reason, the focus of this review article is TGFβ and mainly BMP.

### 2.1. TGFβ family and its signaling

TGFβ, as one of the most abundantly found cytokines in bone matrix, is produced by osteoblasts and found merged in mineralized matrix, even in higher amounts than BMPs [18,24,31,36]. Although its role in osteogenesis has not been explained clearly yet, it is shown to be released during bone resorption to recruit bone marrow mesenchymal stromal/stem cells (BMMSC) to resorption sites, and to limit the further osteoclast formation indirectly by reducing the ability of osteoblasts to secrete RANKL (an osteoclast differentiation factor) and is also reported to promote matrix production, osteoblastic differentiation, yet still noted to stimulate bone resorption by differentiation of osteoclasts [17,18,26,36,37]. During development, growth and fracture healing, it is highly expressed in mature osteoblasts and is relevant to skeletal morphogenesis such as generation of bone shape, bone growth or bone remodeling since it is also effective in bone and cartilage formation [17,24,38,39]. TGFβ is reported to either inhibit or stimulate the osteoblastic cell proliferation depending on the cell densities, species and stage of osteoblast differentiation [17,18,24]. Although TGFβ expression is decreased in human bone with aging, the level of TGFβ is reported to be elevated in bone from patients with osteoarthritis [24]. 

Expression of osteoblastic differentiation markers such as, ALP, Col I and osteonectin are reported to be increased under the effect of TGFβ, while osteocalcin synthesis is reported to be inhibited in human osteoblasts [24]. In contrast, it is reported to inhibit the differentiation of osteoblasts in rat osteoblast cultures and the mineralization via interaction of HDAC4 and HDAC5 recruitment to Runx2 which is promoted by Smad3 connection [24,26,28,36]. Both TGFβ1 and TGFβ2 are, however, shown to promote osteoprogenitor cells into osteoblasts through non-Smad MAPK pathways or Smad 2/3 pathways also [26]. TGFβs are also known as both suppressors and promoters of cancer [29,33]. 

**Figure 5:**
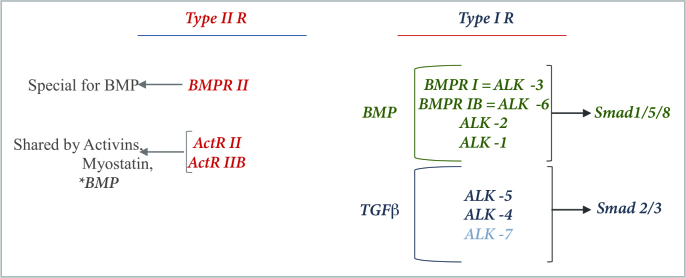
Receptor types in BMP and TGFβ signaling. *BMP has weak affinity for ActR II and ActR IIB.

After posttranslational modifications, TGFβ is secreted as an inactive complex, known as latent TGFβ, which is activated by pH changes or proteases [24]. Decorin or betaglycan binding is reported to be protective against the protease activation of TGFβ [24]. Since osteoblasts are known to produce plasminogen activators, they can also mediate TGFβ production and activation [24]. TGFβ has high affinity for Alk5, Alk4 and Alk7 throughout type I-R and binding causes the activation of Smad 2/3 complex [23, 26, 29, 33] (Figure 5). Activated Smad 2/3 complex binds to Co-Smad (Smad 4) and is recruited to nucleus. For maintaining the normal organization of chondrocytes in growth plate, this Smad 4 mediated TGFβ signaling is reported to be vital [26]. On the other hand, Smad 7 may inhibit the Wnt activity by binding to β-catenin to promote Smurf2 mediated ubiquitination of β-catenin for degradation, which is reported to be an important factor especially for TGFβ induced β-catenin regulated apoptotic responses [25]. 

Because of the complexity of signaling pathways, the relation of BMP and TGFβ is not totally clarified yet. There are a few studies [24,33,36] that reported the regulatory effects of BMP and TGFβ on each other, so that we are able to know the presence of BMP increases the osteoblast TGFβ expression as well as TGFβ auto-induced TGFβ expression in osteoblasts; or TGFβ is known to induce BMP-2 expression in osteoblasts as well as TGFβ-potentiated BMP osteoinductive activities; and not only osteogenesis related events but adipogenesis is also reported to be regulated by the balance between BMP and TGFβ signaling. Both TGF-β and BMP signal through Smad4 [26]. BMP/TGF-β-activated Smads together with Runx2 forms the skeleton [26]. TGF-β and BMP synergistically effect ectopic bone formation [26]. 

The rational changes in type I and type II receptors are also thought to have effects on the differentiation because TGFβ is mentioned to be not capable of binding type I receptors in the absence of type II receptors; while the presence of type II receptors they have only an affinity increasing effect on BMPs [18, 33]. In order to explore the roles of endogenous TGFβ in osteoblast function in vivo, Filvaroff et al. [18] truncated type II TGFβR from the osteocalcin (OCN) promoter to develop a transgenic mouse in which the osteoblastic responsiveness to TGFβ is inhibited. They found an altered responsiveness in cartilage and synovium cells which caused a joint degeneration similar to human osteoarthritis and an increase in trabecular volume in femurs of the transgenic mice by postnatal 35th day and up to months of age the increase in trabecular volume became more pronounced compared to wild type mice even after ovariectomy. They also noted, in transgenic mice the osteocyte density was far lower than the wild type mice which suggests that TGFβ signaling is important for normal osteoblast differentiation in vivo. They concluded that TGFβ has a direct effect on regulation of bone remodeling, structure and biomechanical properties via osteoblasts. In another transgenic mouse study published by Erlebacher et al. [17], TGFβ2 overexpression was analyzed. Increase in osteoblastic and osteoclastic activities were observed with a consequent increase in bone turnover which resulted in a net imbalance between bone resorption and formation, concomitantly a progressive, age dependent bone mass. They also observed mineralization defects in transgenic mice. Both studies above, done with transgenic mouse models can be concluded as, 

1. Type II TGFβR acts as a dominant negative inhibitor of TGFβ signaling, 

2. TGFβ2 may negatively regulate bone matrix mineralization in vivo.

The osteoblastic and/or bone regenerative effect of TGFβ depends on the cell-medium type and the dose/concentration of the growth factor [40]. Centrella et al. [40] mentioned the stimulatory effects of TGFβ on the replication of MSCs in serum free monolayer, however the effects of TGFβ on mitogenic response of some cells to other growth parameters were inhibitory. They also observed the effect of TGFβ on parietal bone cells, CRL1570 cells and CCL92 cells, with two different doses of TGFβ (15 ng/mL and 50 ng/mL) and marked the dose dependent stimulatory effect of TGFβ on DNA synthesis in confluent cultures of osteoblastic cells while it had a slight effect on the osteoblast depleted parietal bone cell cultures [40]. In the same study, it was reported the collagen synthesis was positively correlated with the TGFβ dose while ALP activity was negatively correlated with TGFβ concentration. 

In order to evaluate the enhancement of bone growth with TGFβ1 in a canine model for 4 weeks, Sumner et al. [38] spray coated porous titanium rod implants with HAp (hydroxyapatite) and TCP (tricalcium phosphate) with different doses of TGFβ (335 µg and 120 µg) and implanted them bilaterally to the proximal part of the humerus of each dog with 3 mm gaps between the surface of the porous coating and the host bone. Although bone ingrowth was reported to be measured in both groups, it is mentioned that the amount of bone ingrowth in 120 µg TGFβ1 group was higher than 335 µg TGFβ group and increasing TGFβ dose caused no significant increase in bone growth. In another study [41] done with adult mongrel dogs to observe the dose dependent effects of rhTGFβ2 with 4 different doses as 0 µg/implant, 1.2 µg/implant, 12 µg/implant and 120 µg/implant with HAp/TCP carriers, the implants placed bilaterally in proximal humeri with 3 mm gaps for 4 weeks. They found the local application of rhTGFβ2 strongly enhanced bone ingrowth and gap healing via intramembraneous pathway in a dose dependent manner. They reported 12 µg rhTGFβ2/implant dose was more stimulatory than other dose groups, and excessive doses could be inhibitory by negatively affecting the osteoid mineralization. Elimelech et al. [37] tested the different dose effects of TGFβ with βTCP both in vitro (MSCs with TGFβ doses: 40 ng/mL, 10 ng/mL, 1 ng/mL) and in vivo (rat calvaria mouse model with TGFβ doses: 40 ng/mL, 0 ng/mL). The results showed that TGFβ1 had an inhibitory effect on cell proliferation in a dose-time dependent manner and the maximum inhibition was seen in 40 ng/mL group after 24 h in vitro while it had no effect on cell adhesion to βTCP in any of the dose groups. In vivo, the new bone amount was reported to be almost same within the 0 ng/mL and 40 ng/mL groups. 

### 2.2. BMP family and its signaling

The largest subset in the TGFβ superfamily is represented by BMPs which are actively effective in ectopic and heterotropic bone formation as well as morphogenesis, bone remodeling, fracture repair, proliferation, differentiation, migration, osteoclastogenesis, osteogenesis, stem cell commitment, carcinogenesis, tumor invasion and metastasis, apoptosis, extracellular matrix remodeling, collagen synthesis, immune functions, through direct or indirect mechanisms and can act in endocrine, paracrine and autocrine manner to establish cell and tissue organization [1,8,20,28,31,32,33,39,42–46]. In vitro BMPs are shown to increase ALP expression in osteoblasts and bone mesenchymal stromal cells and to commit MSCs to osteoblastic lineage [24,34,47,48]. Moreover, heterotrophic bone inducing activity is reported to be related with BMPs and growth differentiation factors (GDFs) rather than TGFβs, activins and several BMPs and GDFs of TGFβ family [33]. More than 20 BMPs were defined until now and throughout these BMPs only BMP1, a metalloproteinase, is known to be a nonmember of TGFβ superfamily, yet has a role in collagen maturation as a procollagen C-proteinase and BMP activation as well as bone and cartilage induction. [33,34,36,45,49,50]. In addition, BMPs’ osteoinductive activity is reported to be significantly higher than TGFβ [24]. 

**Figure 6:**
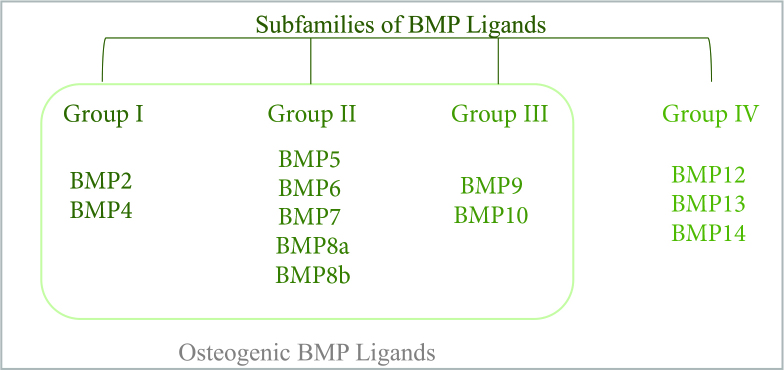
BMP ligand subfamilies due to their amino acid sequence homology, structure and functions.

Contrary to TGFβ, BMPs are not secreted as latent inactive forms [24] and depending on their amino acid sequence homology, structures and functions BMP family can be divided into 4 subclasses as subclass I (BMP-2, BMP4), subclass II (BMP5, BMP6, BMP7, BMP8a, BMP8b), subclass III (BMP9, BMP10), and subclass IV (BMP12, BMP13, BMP14). However, throughout these BMPs, only BMP2 to BMP10 (except BMP3 which is an inhibitor as BMP13) can be classified as bone inducing BMPs, which can also be divided into 3 subgroups as Group 1 (BMP-2, BMP4); Group 2 (BMP5-BMP8) and Group 3 (BMP9, BMP10) depending on their amino acid homologies [28,34] (Figure 6).

**Figure 7:**
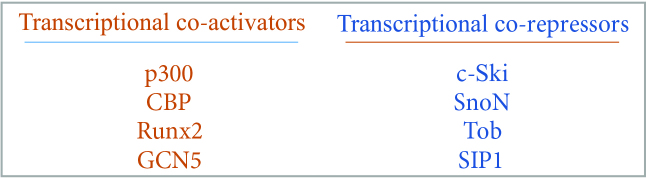
Transcriptional coactivators and coregulators.

The canonical BMP signaling pathway is reported to be evolutionarily conserved over at least 700 million years [28]. In Smad dependent canonical pathway, BMP regulation occurs from extracellular space through nucleus via type I and type II receptors, which can be named as BMP Receptor I (BMPRI) and BMP Receptor II (BMPRII) in BMP signaling. BMPRII is a specific receptor only for BMP ligands and however BMP ligands can also bind to activin receptor (ActR) II and ActR IIB like activins and myostatins [23,28,33]. Ligand binding to type II-R activates the Smad 1/5/8 signaling pathway [22,23,28,29,33]. The activated Smad 1/5/8 makes a complex with Co-Smad, and is recruited to nucleus. In nucleus, they associate with either transcriptional coactivators as p300, CBP, Runx2 and GCN5 or corepressors as c-Ski, SnoN, Tob and SIP1 and bind to regulatory elements of the target genes for transcriptional regulation [28,32,33] (Figure 7). In nucleus, the interaction with coactivators p300 and CBP are reported to be important for the transcriptional activity of phosphorylated R-Smads [33].

Runx2 is a key transcription factor in osteogenesis and indispensable for bone formation [26,32]. Smad1 is reported to interact with Runx2 on the promoter of target genes to control osteoblastic gene expression and differentiation [26]. Li et al. [32] also reported that although Runx2 has been shown to interact with TGFβ-specific Smads (Smad 2 and Smad 3) to block myogenic differentiation via mimicking the common effects of TGFβ and BMP-2, it only synergizes with BMP-2, but not with TGFβ, to induce osteoblastic differentiation. 

 Depending on the information given above, it is not hard to see otherwise the Smad 1/5/8 pathway might be halted without phosphorylation of R-Smads and the interaction of Smad1/5 with Runx2 – a key transcription factor in osteogenesis – would be distracted. 

Smad 6, as an I-Smad, also takes part in negative feedback mechanism of BMP signaling and it is required to limit BMP signaling during endochondral bone formation and is recruited to cytoplasm from nucleus via Smurf1 binding; because it is mainly in rest state in nucleus. Smad 6 binds to Smad 4 to form a nonfunctional complex in order to halt the binding of Smad 1/5/8 to Smad4 and inhibits the BMP signaling in bone formation [26,32]. Chen et al. [26] mentioned about the conditional deletion of Smad 4 in osteoblasts lead to lower bone mineral density, bone volume and decreased the bone formation rate and osteoblast number.

Although BMP is one of the most popular growth factors which are known as triggering / signaling molecules that regulate not only growth but also repair and regeneration [1,21] by promoting osteogenesis, chondrogenesis, vascularization and formation of fibrotic tissue to accelerate the maturation progress and callus formation while inhibiting osteoclastogenesis [8], decreasing blood loss, surgery time and hospital stay [20,21]; the use of BMPs is still avoided by many physicians because of their complications such as an increase incidence in dysphagia, significant prevertebral swelling, airway edema compromise [1,2,20,51], uncertain impacts in promoting tumorigenesis and tumor metastasis [45,52], ectopic bone formation, osteophyte production [1,20,43,44]. It is also reported that BMP-2 induces PPARγ activation in a dose dependent manner and promotes the commitment of MSCs to adipocyte lineage [20,53]. Thus, it is important to use BMP in certain amounts with proper delivery vehicles for dosage control and sustained release and to lengthen the half-life of the cytokine in body [9,10,54]. For this purpose, BMPs are used with many natural or synthetic polymers or composites in the literature (Table).

**Table:** Table. Carriers of the BMP molecule

Ref.	GF/Material	Experiment model	Results
55	BMP+OPF-BP hydrogel with PLGA microspheres	Rat subcutaneousimplant model	BMP absorbed on the phosphorylated hydrogel showed better results than the ACS (absorbable collagen sponge control) group and the boneformation was 12-fold higher.
56	rhBMP-2 (0, 6, 9, 12 mg per side)+HA:TCP (60:40)	Rhesus monkey – L4/L5 laminectomy BLIPA(Nonhuman primate lumbar intratransverse process arthrodesis) model	In control group (AIC-Autologous iliac crest) and BMP 0 mg group no fusion is observed. The 6 mg, 9 mg and 12 mg groups showed solid fusion with 9 mg and 12 mg rhBMP-2 groups resulting in a formation of quantitatively more and denser bone.Bone formation in 12 mg groups reported to be not much than 9mg groups.
49	BMP-2+HA (Hyaluronic Acid)+hMSCs	Rat calvarial defect model	Within different combinations of BMP-2-HA-hMSCs, highest OSN expression and mature bone vascular markers were observed in HA + hMSCs + BMP-2 group within 4 weeks
57	IGFβ1/TGFβ1/BMP-2+Ti-discs coated with PDLLA	Sheep musculus cleido mastoideus implantation model	No ectopic bone formation was observed in any groupsCapsule formation was observed only in the GF (growth factor) loaded side, the GF application influenced the proliferation of the fibrous tissue.
58	hBMP-2+ Opencell PLA (OPLA)	Beagle L4-L5 postero-lateral spinal fusion model(implantation on transverse processes)	BMP + OPLA combination was shown to be superior to AIC.By 3 months 100% fusion was observed in BMP + OPLA groups while no fusion was observed in AIC only groups.
59	rhBMP-2(96 µg, 48 µg, 0 µg)+PLA	Rat nonhealing defect in mandible	At 13–26 weeks the bone production between 0 µg and 48 µg groups were not significantly different.although the thickness of the bone was observed in 48 µg group, it decreased in time.96 µg group showed the most bone formation and the thickness of the bone tissue was maintained.
60	BMP-2+PEG-based hydrogel (OPF. Oligo PEG fumarate with BP-bisphosphonate)	Rat femoral defect model	Phosphate functionalized on BMP-2 surface enhanced BMP-2 induced ectopic bone formationPhosphate functional groups enhanced the osteoconductive characteristics of OPF
61	GF1/BMP-2+OPF	Rabbit knee defect model	Combined application of IGF1 + BMP-2 resulted in better subchondral bone repair than IGF1 group itself.BMP-2 alone resulted better bone score morphologyDelivery of BMP-2 is thought to accelerate the bone formation at an early time point.
62	BMP-2+Gelatin-βTCP Sponge+MSCs	Rat subcutaneous implant model	Differentiation of MSCs is enhanced by BMP-2 in vivo and BMP-2 was retained in sponges more than 4 weeksTissue maturation, increased new bone volume and ALP, OCN expressions were observed.
63	BMP-2 (0 µg – 0.08 µg – 8 µg per 1mg of PLGA microspheres)+BMSCs+PPF (propylene fumarate)	Goat ectopic implant model	Compared to BMP impregnated scaffolds, microsphere implantation caused a lower burst release and sustained over a lone period of time. No significant effect of BMSC addition before implantation was reported on the osteoinductive capacity.
64	BMP-2 (6 ng, 60 ng)+Gelatin vs PLGA microparticles +PPF scaffolds	In vitro release profile experiment	BMP-2 release from gelatin microparticles happened with a minimal burst release and linear release kinetics afterwards.PLGA microspheres showed a moderate BMP-2 burst release followed by a minimal cumulative release profile.Addition of collagenase also increased the release of BMP-2 from the scaffolds
65	rhBMP-2+Chitosan (CS) microspheres	Rat muscle bag model	Enhanced ectopic osteogenesis and promoted osteoblast differentiation by enhancement of ALP activity and calcium content
66	BMP-2 (0 µg, 1 µg, 10 µg)+Collagen sponge	Mid-diaphysis full defect model in old vs young rats	Increased expression of critical genes were observed to decrease to their baseline levels after 4 weeks in young rats while many genes including osteoblastic and osteoclastic genes, remained upregulated in old rats up to 6 weeks 1 µg BMP-2 exhibited prolonged inflammation compared to 10 µg groups which might be related with the poor healing of these groups.
67	rhBMP-2(0 µg, 1 µg, 5 µg, 10 µg, 20 µg)+Collagen Sponge	Rat femoral defect model	Defects with higher doses of rhBMP-2 showed increased bone bridging in the gap however the remodeling level was reported to be same for 10 µg and 20 µg groups.The radiographic score increased with increasing BMP dose.However 20 µg group showed lesser healing compared to 10 µg group
68	BMP7+PLAGA [poly(lactide-co-glycolide)] matrix	Rabbit skeletal muscle cells	Enhanced synthesis of OCNIncreased calcium and phosphate depositionDifferentiation of skeletal muscle cells to osteoblast like cell profile

There are also studies done with metallic mega-prostheses for large bone defects however they are reported to have higher complication rates than conventional arthroplasty and may require revision surgery due to infection, loosening and increased wear particles which may disperse along the joint space in some cases and can also occupy the adjacent tissues and cause a shift in homeostasis of bone tissue to osteolysis over time [11,69].

Koolen et al. [19] used commercially available fibrin as a carrier for BMP-2 in vitro with ATDC5 cells and in a femur CSD (critical size defect) model in Wistar rats and observed a burst release of BMP-2 from the constructs which continued for 28 days with ALP activity in vitro. In vivo they observed an increase in bone formation with defect bridging and remodeling without a trace of ectopic bone formation. Chen et al. [70] also used HLC (human like collagen) as a carrier for BMP-2 because of its high affinity for BMPs and its being a natural part of human bone, in vitro (on SD Rat MSCs with 1 µg BMP) and in vivo (on Kunming mice as ectopic bone formation model, and on SD Rats as calvarial defect model with changing concentrations of BMP as 0 µg, 1 µg, and 5 µg with implant and without implant). In vitro they reported an increase in Runx2, Alp and OPN (osteopontin) protein and gene expressions. In vivo, although no ectopic bone formation in the absence of HLC implant was observed, the ectopic bone formation was seen in all groups with BMP + HLC implants. In rat calvarial defect model, the bone formation was increased with the increasing doses of BMP, however in 5 µg BMP + HLC group a significant bone overgrowth was observed as side effect and 1 µg BMP + HLC group was reported to be the only effective group with no side effects. Kaito et al. [2] combined interconnected porous hydroxyapatite (IP-CHA) and PLA-PEG (polylactic acid - polyethylene glycol) composites as a carrier system for different rhBMP-2 doses (0 µg/mL, 5 µg/mL, and 20 µg/mL) and investigated the bone repair capacity on CSD in rabbit radius. The results at 8 weeks postoperation showed 5 µg/mL and 20 µg/mL rhBMP-2 groups had almost same results histologically, biomechanically and radiologically and they reported a decrease in the required amount of rhBMP-2 for bone regeneration in rabbit radius CSDs.

The composites or biomaterials that are developed to provide a sustainable release of BMP or let us say, cytokines, are of course must be confirmed by the preclinical studies because in vivo, ex vivo and in vitro effects of the materials or cytokines most probably will be different. However, from an ethical point of view, in order to decrease the number of experimental animals used in preclinical studies and/or in order to provide more quantified release results, first these composites or biomaterials should be tested in vitro for release kinetics and/or cell studies before going for animal trials to see if the released cytokine amounts and/or the biomaterials/composites are suitable and if the release is sustainable and the released cytokines preserve their activities. To observe the release activity of a cytokine from the composite there are many methods reported such as Elisa [71], radiolabeling of the cytokine [72,73], colorimetric assays as BCA [74]. Depending on the results of these assays, the cytokine, composite or biomaterial can or cannot go for other in vitro cell tests and/or animal experimenting and by using these release kinetic tests the time-dose dependent results of the cytokine released from the composite can be obtained and the activity of released cytokines from the composites can either be tested in vitro, in vivo or both.

## 3. Clinical studies

There are also various applications of BMPs and TGFβs in clinics with carrier molecules [51, 75–84]. However, the locally applied supraphysiological BMP-2 doses during surgery is reported to be connected with hematoma formation in soft tissue, increased inflammatory response, bone cysts and infection [85] which also increase the importance of clinical follow-up studies.

Burkus et al. [82] compared the application of rhBMP-2 with collagen sponge or with autologous iliac crest bone in a total of 279 patients in 2 years follow up study after interbody fusion using two tapered threaded Ti-fusion cages and reported a higher fusion rate in rhBMP-2 with collagen group.

Since BMP is reported with side effects, Maza et al. [51] observed the results of small sponge rhBMP-2 application either within an allograft or a PEEK cage in 47 patients between 33–74 years of age spectrum within 2010 January and 2016 November and reported no incidence of expected complications such as dyspnea, edema, ectopic bone formation, or life threatening respiratory events such as prolonged intubation or complications referred to steroid usage as delayed healing or diabetes. Sebastian et al. [77] also aimed to evaluate the safety and efficacy of BMP-2 use in transforaminal lumbar interbody fusion (TLIF) with regard to postoperative radiculitis in 77 patients between 18–75 years of age and reported that not only TLIF with BMP use did not lead to postoperative radiculitis but also an improvement in back pain was seen in patients. 

Similar to preclinical studies, in clinical studies the dose dependent effects of rhBMP-2 were also observed. Lytle et al. [78] reported, in the applications of transforaminal interbody fusion between the years 2009 to 2014, there observed an increase in fusion odds as BMP dose increased from 0.16–1.0 mg/level to 1.0–2.0 mg/level while no increase in fusion odds was observed when BMP dose was more than 2.0 mg/level and reported that 1.0 mg/level was the minimally effective dose and concluded that as the BMP dose/level is a significant precursor of fusion. Govender et al. [75] also reported a 20 months follow up study of an open tibial fracture case of 450 patients with study groups of standard care (SC–intramedullary nail fixation and routine soft tissue management), SC + implant (absorbable collagen) coated with 0.75 µg/mL rhBMP-2 and SC + implant coated with 1.50 µg/mL rhBMP-2. They observed a 44% decrease in the risk of failure, and increase in fracture healing time and wound healing time in SC + implant coated with 1.50 µg/mL rhBMP-2 group compared to SC.

These results may show us that, the required dose of BMP-2 also depends on the application place, the surgical procedure as well as the implant type.

## 4. Perspective

Pseudo-arthrosis which may result from complete fractures, huge bone gaps as a result of tumor resection, need for the large amount of trabecular bone for spinal fusion surgeries, osteonecrosis as well as impaired bone healing are the main problems in orthopaedics [86,87]. In clinics, open fractures, tumor resection, insufficient immobilization, inadequacy or lack of blood supply, poor nutrition or the effect of other metabolic diseases such as diabetes, may cause delayed healing or nonunion or the decrease in bone mineral density with age and osteopenia and/or osteoporosis may cause fracture, nonintegration of the material/instrument or collapse of the surgical procedure and/or composite [1,11,86,88,89]. Lee et al. [90] reported the need for bone grafting is between 0.5–1.5 million per year in the USA alone and half of them are for spinal fusion surgeries yet the pseudo-arthrosis is reported to be 5%–43% for posterolateral spinal grafting [90]. Also, the delayed healing rate was reported to be in the range of 16%–60% for less severe fractures and 43%–10% for more severe fractures and the nonunion rate is reported to be 4%–10% [89]. In addition to postoperative problems or complications, the economic side of these surgeries and instrumentations as well as composites is another problem that we have to face in clinic [79,91].

It is well known that for the integration of the material the osteoinductivity, osteoconductivity and osteogenic activity are important. However, in patients with osteoporosis, the increased bone remodeling and the negative final bone balance based upon the decreased osteoinductive, osteoconductive and osteogenic capacities of the bone, becomes one of the main problems related with the poor bone fusion [88]. Diaz-Romero Paz et al. [88] mentioned the other scoliotic deformities are also seen in high percentages as 36%–48% in women with osteoporosis and spinal surgery complications are mostly seen in patients aged over 65 or with osteoporosis. In spinal fusion, the instruments used, such as screws or nails, as well as the bone that they are inserted in are exposed to body fluids and stress, which may increase their corrosion. The bone area around the implant will be affected by this corrosion effect and especially in cases with decreased bone mineral density (BMD) as it is seen in osteoporosis, this damage in and around the implant will be drastic. The pullout or removal of the pedicle screws are reported to be one of the most seen complications within 3 months after spinal surgery [88]. Hence, a suitable material that can contribute an increase in the BMD values at the area of application of instruments while providing bone formation concurrently, seems essential in spinal or other orthopedic applications. 

Bone growth factors are the cytokines that naturally take part in development processes as well as new bone formation, fracture healing, proliferation and differentiation [20,45,90]. Throughout them, TGFβ superfamily as previously mentioned is one of the most popular because of its effects on bone. Within this family, BMPs as cytokines with the highest osteogenic properties, are the ones which are studied widely in bone regeneration applications as well as in clinic and within them, rhBMP-2 is reported to be the first BMP that was introduced as a bone graft substitute [20]. After the approval of its use in clinics, the use of rhBMP-2, especially in spine surgeries, increased drastically because its use decreased blood loss and surgical time and increased fusion rates [1,21,81]. However, the increased use of rhBMP-2 also brought the negative side effects depending on the high dose usage are also mentioned in this article previously, since they are already pleiotropic [43], yet these are not the only problems related with BMPs. Solubility of BMP is also one of the major problems to be overcome before the clinical applications [46,54] because the fast release of BMP will result in the loss of the cytokine in the application area and will decrease the regenerative effect of it. Nevertheless, these complications did not prevent the use of these cytokines in the clinics because the clinical studies showed better results with BMP-2 than autogenous bone grafting alone [20,92]. The advantage of increased rate in spinal fusion with the combination of BMPs [20,56,76,78,79,91] and BMPs’ having high capacity for bone regeneration and capability of fracture healing keep them popular in bone regeneration studies and make them a popular research area also in basic science in order to find suitable carriers for these cytokines to be released in a sustained manner with a decrease in required dose for bone regeneration. Thus, it is an important research area to find new materials to fill bone defects and/or to support the instruments that are used to stabilize the bone while supporting bone regeneration and the sustained release of the cytokine and/or to understand the signaling pathways of cytokines, how they work in cellular level on differentiation and proliferation in order to prevent the probable progression of a disease before it needs a replacement surgery. 

In addition, the dual effects of both TGFβ and BMPs necessitate the better understanding of the molecular pathways of the growth factors that are going to be used in bone regeneration studies. As an example, TGFβ is known to be related with not only bone formation but also tumorigenesis and in tumorigenesis it is reported to have a dual role as a tumor suppressor in early stages of carcinogenesis and as a promoter of tumor metastases in advanced stages of carcinogenesis [93]. In addition since TGFβ, as mentioned before, has pleiotropic effects, it is not only related with bone but its activity is also observed in ovarian, pancreatic, colon cancers and in squamous cell carcinoma and is reported that the over activity causes Camurati-Engelmann disease of bone, which occurs because of a missense mutation in the latency associated peptide that causes a constitutively active TGFβ [94]. Similarly, a gain of function mutation in Alk-2, as one of the BMPRI, causes fibrodysplasia ossificans progressiva (FOP) [33]. Previously in this paper, BMPs’ and TGFβs’ probable effects on EMT were also mentioned. Although in this paper mostly Smad pathways are mentioned, for TGFβ to induce the EMT a cooperation between Ras/MAPK and TGFβ/Smad pathways is required [93] which shows us the complicated relation between signaling pathways of molecules should to be studied and understood further not only for the successful and suitable applications of cytokines with no or low side effects for bone regeneration but also for the treatment or prevention of the genetic bone diseases related with these cytokines. 

## 5. Conclusion

Proteins in TGFβ super family are all growth and differentiation factors that take role in organogenesis, tissue remodeling and survival [45]. BMP is one of the biggest subfamilies of TGFβ superfamily and more than 20 human BMPs are described until now [20,45]. They are known to take part in bone formation, cell proliferation, migration and survival [23,34]. It is also mentioned that Klf4 as one of the factors with Oct4, Sox2, and c-Myc, which are important for redifferentiation of mouse embryonic fibroblasts to mouse ES-like stem cells (iPS Cells) and can be replaced by BMPs which is a proof of their influence in MET (mesenchymal epithelial transition) and shows their significant roles in the maintenance and differentiation of pluripotent stem cells [33].

 Despite their positive effects on bone regeneration, the complications restrict the use of these growth factors. The most common adverse effect of BMP-2 use is defined as ectopic bone formation [20], however they are also implicated in triggering epithelial phenotypic changes that cause them become more similar to mesenchymal cells [45], which increases the probability of epithelial mesenchymal transition – a cause of metastatic potential in oncogenic EMT. Similarly, TGFβ activation is shown to be activated in an EMT related Snail1 gene and resulted in the repression of the transcription of e-cadherine gene [28]. In basic biological studies, upregulation of BMP ligand expression has been shown in various carcinomas, and also they have been mentioned to increase the invasiveness of prostate, lung, oral SCC and breast carcinoma as well as the proliferation within multiple carcinoma types [20]. Katagiri et al. [33] reported TGFβ and BMP are both suppressors and promoters of cancer and mentioned the importance of autocrine TGFβs in maintenance of stem cell like properties and tumorogenic activity of glioma initiating cells (GICs) and the effects of BMP signals on induction of differentiation of GICs. Yet, there are other studies emphasizing the increased expression of BMPRII has a suppressive role in tumors [95] which brings us to the result that the effects of BMP on tumor formation or carcinogenesis should be dose dependent. 

BMPs and TGFβ take part in bone homeostasis and regeneration together, however the relation between TGFβ itself and BMP on bone regeneration process has not been clearly understood yet. Asparuhova et al. [96] analyzed the TGFβ and BMP-2 protein release from mesenchymal stromal cell line ST2 on deproteinized bovine bone mineral (DBBM) and collagen membranes precoated with bone conditioned medium (BCM). BCM was reported to induce enhanced proliferation of TGFβ1 and BMP-2 specific R-Smads and TGFβ showed faster release kinetics than BMP-2. They also examined TGFβ1 effect on BMP with various concentrations of TGFβ1-only or various concentrations of TGFβ1 combined with certain dose of BMP-2. In all groups BMP alone was shown to upregulate the expression of all osteoblast specific mRNAs. TGFβ1 effect was also reported to increase the Colα1, Colα2 and Spp1 mRNAs while it slightly decreased the mRNA expressions of Runx2, Bglap2, Ibsp, and Alp1 in a dose dependent manner so they concluded that TGFβ1 exhibits a stimulatory and dose dependent effect on BMP-2 induced osteoblast differentiation. The mineralization was reported to be stimulated with BMP-2 but not TGFβ1, however TGFβ1 was shown to enhance BMP-2 induced deposition of mineralized matrix by ST2 cells significantly. They also proved the stimulation in Smad1/5/8 pathways was seen 24 h after BMP-2 introduction although the levels of Smad1/5/8 was decreased after 2 h. However, with combination of TGFβ1 and BMP-2, Smad1/5/8 pathway was also stimulated and the expression was prolonged compared to BMP-2-only introduction. It was also reported that the presence of BMP increased the osteoblastic TGFβ expression or vice versa and TGFβ was shown to potentiate the osteoinductive activities of BMPs which suggested a positive feedback mechanism between these two growth factors [24]. 

For cytokines with pleiotropic effects and complicated signaling pathway relations which have not been understood completely yet, the use in clinics will also bring the complications regardless of the positive effects on bone or cartilage regeneration. For growth factors to be effective, the cytokines should be kept in the implantation site to ensure they will be able to exert their biological actions [9]. To provide this, the development of new biomaterials which are proper not only as carriers for sustainable release of cytokines but also as cell substrates, nutrient suppliers, or mechanical supports is as important as the study of signaling pathways between cytokines and/or related diseases. 

**Conflict of interest**

All authors do not have any conflict of interest.
